# Characterizing patients who benefit from mature medical AI models in real-world clinical applications

**DOI:** 10.1371/journal.pdig.0001283

**Published:** 2026-03-20

**Authors:** Zhiyi Chen, Wei Li, Zhicheng Lin

**Affiliations:** 1 Experimental Research Center for Medical and Psychological Science (ERC-MPS), School of Psychology, Third Military Medical University, Chongqing, China; 2 The Chinese Open Science Network, Nanjing, China; 3 School of Psychology, Southwest University, Chongqing, China; 4 Key Laboratory of Cognition and Personality, Ministry of Education, Chongqing, China; 5 Department of Psychology, School of Humanities and Social Sciences, University of Science and Technology of China, Hefei, China; 6 Department of Psychology, Yonsei University, Seoul, Republic of Korea; National Tsing-Hua University: National Tsing Hua University, TAIWAN

## Abstract

Medical artificial intelligence (AI) is being rapidly deployed in clinical practice, yet its real-world effectiveness across diverse patient populations remains poorly characterized. We conducted a systematic review combining automated screening (fine-tuned BERT-PubMed classifiers) with manual validation to identify studies of mature medical AI models deployed in healthcare facilities worldwide. We included 171 studies at the “device-into-practice” stage with sufficient demographic and performance data, representing 209,772 patients. Patient access to these models showed marked demographic disparities: geographic concentration was extreme (Dagum–Gini coefficient 0.97, *P* < .001), with 95.1% of patient cohorts (studies) from high-income (62.2%) or upper-middle-income (32.9%) countries—primarily China (28.7%) and the United States (18.9%)—and no studies from low-income countries. Racial representation was dominated by White (49.1%) and Asian (42.6%) patients, and 63.8% of studies exhibited moderate-to-high sex imbalance. Across all studies, AI models outperformed human practitioners (81.7% vs. 77.8% accuracy, *P* < .001), but this superiority was confined to in-distribution applications (same geographic/demographic context: 82.9% vs. 77.3%, *P* < .001) and disappeared in out-of-distribution deployments (cross-geographic/demographic contexts: 74.1% vs. 76.3%, *P* = .45). In underrepresented populations, AI performance was not significantly different from that of human practitioners. Overall, mature medical AI models are deployed predominantly in economically advantaged settings, with performance advantages concentrated in well-represented demographic groups, highlighting a digital divide in access and effectiveness, and the need for demographic-specific validation.

## Author summary

Artificial intelligence (AI) is rapidly transforming healthcare, but it remains unclear who actually benefits from these technologies in everyday clinical practice. We systematically reviewed 171 studies involving over 200,000 real-world patients and found that access to mature medical AI models is heavily skewed: 95.1% of patient cohorts were from high- or upper-middle-income countries, primarily China and the United States, with no representation from low-income countries. Most evaluations focused on White (49.1%) and Asian (42.6%) patients, and nearly two-thirds of studies showed substantial sex imbalances. Across studies, AI models generally outperform human practitioners on diagnostic and prognostic tasks, but this advantage was seen only when systems were used in populations similar to those in which they were developed. When deployed in different regions or demographic groups, AI performance decreased and was no better than that of human clinicians, while typically remaining comparable rather than clearly worse. These patterns challenge the idea that medical AI systems are universally applicable and reveal a digital divide in who benefits from them. Our findings highlight the need for more inclusive AI development, routine monitoring of performance after deployment in diverse populations, and, where appropriate, region-specific adaptation of models so that AI can support patients more equitably across different settings.

## Introduction

Artificial intelligence (AI) in medicine has garnered widespread enthusiasm from scientific and commercial sectors, yet its real-world effectiveness is increasingly questioned amid a clinical translational crisis [1,2]. This crisis reflects premature, inequitable, or invalid model deployment that can harm patients, particularly among vulnerable groups [3]. Although existing critiques document systematic biases arising from economic disparities and uneven biomedical data distribution during model development, how these biases affect generalizability and clinical utility once systems are implemented remains unclear [1,2,4]. Despite these unresolved issues, regulatory approval and clinical deployment of AI models continue to accelerate, raising pressing questions about whether benefits are equitably distributed in routine clinical practice. At the same time, the absence of systematic post-market evaluations of real-world performance deepens concerns about a widening gap between development-stage promises and actual clinical outcomes [5,6].

Current assessments of medical AI utility in diagnostic, prognostic, and predictive applications are constrained by data sources that draw largely from FDA-regulated devices in the United States (U.S.) and retrospective electronic health record analyses that often lack comprehensive patient demographic information [5,6]. Although recent research has documented geo-economic and sex/gender disparities in clinical studies of AI-enabled applications [7], there remains a lack of systematic post-market evaluations of model performance across diverse populations in everyday clinical practice [8,9]. Consequently, it remains unclear which specific patient cohorts benefit from or are adversely affected by these models in real-world deployments.

Recent studies have examined particular aspects of AI bias, including racial disparities in cardiovascular applications [10], generative AI diagnostic accuracy [11], and model transfer across hospitals and income settings [12]. However, no systematic evaluation has quantified access to clinically deployed AI models across patient populations globally. Previous reviews have primarily focused on research-stage AI [13] or examined bias thematically [14], leaving the demographic profiles of patients using deployment-ready systems largely uncharacterized. This gap is critical because understanding real-world access patterns is a prerequisite for addressing equity in healthcare.

To address this gap, we examine mature medical AI models—those actively deployed in clinical settings such as hospitals and healthcare centers. These models represent the “device-into-practice” phase within the AI maturity framework, allowing us to move beyond identifying developmental biases to evaluate real-world clinical performance [15]. Using a BERT-based end-to-end natural language processing pipeline, we identified studies evaluating mature AI models used in diagnostic, prognostic, and predictive applications. Specifically, we asked: (1) Which patient populations are accessing mature AI models in real-world clinical settings? (2) How does model performance vary across diverse sociodemographic groups? By focusing on deployed systems rather than controlled experimental settings or theoretical bias, our approach captures the actual integration of AI into healthcare delivery.

## Materials and methods

### Ethics statement

This systematic review followed PRISMA 2020 (Preferred Reporting Items for Systematic Reviews and Meta-Analyses) to ensure transparent and reproducible reporting. The Institutional Review Board of the Faculty of Psychology at Southwest University exempted this study from ethical review as it involved no original data from human participants.

### Literature retrieval

We searched MEDLINE for studies of mature medical AI models published between January 1998 and January 2024 (search completed in January 2024). To capture real-world clinical applications involving prediction, diagnosis, and other quantitative medical decision-making, we used a broad Boolean strategy based on MeSH (Medical Subject Headings) terms: (“artificial intelligence”) OR (“deep learning”) OR (“machine learning”) OR (“neural net”) OR (“supervised learning”) OR (“unsupervised learning”) OR (artificial intelligence[MeSH Terms]).

### Inclusion and exclusion

To identify eligible studies from the retrieved literature, we used the “Global Clinical Artificial Intelligence Dashboard” (https://aiforhealth.app), an end-to-end NLP tool [15] that applies three fine-tuned BERT-PubMed classifiers optimized with AdamW [16]. Studies were included if they met all of the following inclusion criteria (see S1 Method for details):

1. Reported AI models designed to support medical decisions for human patients via prediction, diagnosis, or other quantitative outputs.
2. Evaluated AI models at the “device-into-practice” maturity stage, defined as models approved for or actively deployed in real-world clinical settings.
3. Provided sufficient information on model performance (e.g., accuracy) and patient characteristics (e.g., sex, sample size, geographic origin).

We excluded models used solely for imaging preprocessing, feature extraction, or other ancillary functions; models not tested on human participants; and models that did not meet the required maturity stage. Reviews, meta-analyses, perspectives, and other publications without original data were also excluded.

### BERT-PubMed inclusion classifier

For the first inclusion criterion, a fine-tuned binary BERT-PubMed classifier was used to identify studies reporting AI models that produce predictive, diagnostic, or otherwise quantitative outputs relevant to patient care [17]. The classifier was refined with the question: “Does this model output have a direct, actionable effect on patient care by providing information to a healthcare provider, patient, or automated system?” Studies classified as positive were then manually reviewed to confirm eligibility.

The inclusion classifier was trained using a 9:1 train–test split by Zhang et al. on 4,000 manually labeled PubMed abstracts (1998–2020)—i.e., meeting vs. not meeting inclusion criteria—achieving accuracy and sensitivity greater than 0.98 [15]. Its generalizability was evaluated on 1,000 additional abstracts published after September 2021 and an independent set of 446 abstracts from a systematic review [18]. The training and testing datasets are available in a public GitHub repository (https://github.com/whizzlab/health_ai_training).

### BERT-PubMed maturity classifier

To operationalize the second inclusion criterion, we used the “BERT-PubMed Maturity Classifier,” which identifies AI models at the “device-into-practice” stage—those with regulatory approval or active clinical deployment. This classifier was trained on 2,500 labeled abstracts (1998–2020) [15] and validated on 2,494 more recent abstracts, with additional sensitivity testing on 83 abstracts from a separate systematic review [18]. It achieved accuracy above 0.99 and sensitivity above 0.97.

### BERT-PubMed multi-label classifier

After eligibility was confirmed by the inclusion and maturity classifiers, we applied the multi-label BERT-PubMed classifier developed by Zhang et al., which incorporates a named-entity recognition layer to extract model characteristics, including clinical specialty, target diseases, algorithms, and input modalities [15]. This classifier was initially trained on 4,000 PubMed abstracts and refined with manually annotated labels [19]. Studies for which key characteristics could not be extracted were flagged and excluded.

### Data extraction and validation

All studies identified by the automated pipeline were manually reviewed by the authors to verify eligibility, and GPT-3.5 was used only for preliminary classification (S1 Table). Only “device-into-practice” models with sufficient demographic and performance data were retained for analysis. For each included study, we manually extracted publication details, technical characteristics, patient-level sociodemographic variables (e.g., sample size, sex, country, city, race), and clinical performance metrics (e.g., accuracy for predictions or diagnoses).

Sex representation was summarized using the minority-to-majority sex ratio, categorized as low (0.7–1.0), moderate (0.3–0.7), and high (0–0.3) disparity, consistent with established thresholds for progressive gender imbalance [7]. Racial categorization (“Black,” “White,” “Asian,” “Multiracial”) was derived from self-reported data. When not explicitly reported, race was inferred from the country of origin by mapping World Health Organization (WHO) regional offices classifications (https://www.who.int/about/who-we-are/regional-offices) to the corresponding U.S. Office of Management and Budget (OMB) standards (https://www.census.gov/topics/population/race/about.html). National economic status followed the World Bank 2024 income classifications (high, upper-middle, lower-middle, or low-income; https://databank.worldbank.org/source/world-development-indicators/). Classification accuracy (defined as the proportion of total correct predictions) was used to quantify AI performance relative to human experts. Inter-rater reliability for categorical variables was assessed via independent double-coding, yielding a Cohen’s kappa of 0.70 (*P* < .001), indicating substantial agreement; continuous variables were verified by manual review.

### Statistical analysis

Geospatial models were generated using the maps package in R (version 4.2.2; R Foundation for Statistical Computing, Vienna, Austria) and EasyShu (version 3.6; ES Group, China). Maps of developer and patient distributions were harmonized using the United Nations Statistics Division (UNSD) codes, covering 251 countries, regions, or areas. The authors maintain a neutral stance regarding any territorial disputes, boundaries, or geopolitical classifications implied by these visualizations.

To quantify sociodemographic disparities in the patient populations represented in mature medical AI deployments, we calculated Dagum–Gini coefficients across geospatial, racial, and economic strata, using the number of patient cohorts as the unit of analysis. Coefficients range from 0 (perfect equality) to 1 (perfect inequality), with values above 0.6 indicating substantial between-group disparities [20]. Statistical significance was assessed via a non-parametric permutation test (10,000 permutations). Under the null hypothesis of no systematic group-based disparity, group labels were randomly shuffled to generate an empirical distribution of Dagum–Gini coefficients; the p-value was defined as the proportion of permuted statistics greater than or equal to the observed coefficient. Statistical computations were performed in MATLAB (MathWorks, Inc., Natick, MA, U.S.).

To compare overall performance between mature medical AI models and human practitioners, we used two-sided paired-sample t-tests (α = 0.05), because all AI–human comparisons were based on the same datasets or case sets within each study. For comparisons of AI performance across independent model conditions (e.g., high-cost vs. low-cost input features) and across sociodemographic categories (e.g., high-income vs. lower-middle-income countries), we used independent-samples *t*-tests; when Levene’s test indicated unequal variance, Welch’s *t*-test was applied instead. All statistical analyses were conducted in JASP (0.18.0.1, JASP Team).

## Results

### Selection of studies and sample characteristics

**Fig 1:**
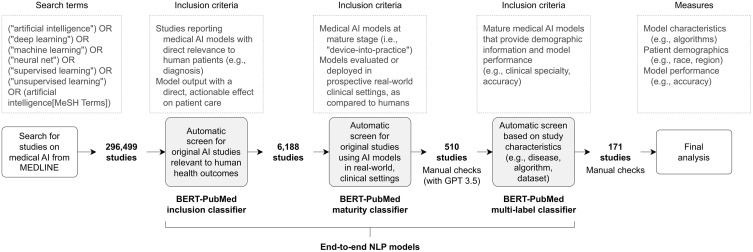
Schematic diagram illustrating the use of the end-to-end NLP model for automated searching, screening, identification, and data extraction.

Of the 296,499 MEDLINE articles reporting AI models between 1998 and 2024 from the Global Clinical AI Dashboard (PRISMA flowchart in S1 Fig), 6,188 studies were identified by the fine-tuned BERT-PubMed inclusion classifier as focusing on medical AI models developed for clinical decision support. Subsequent evaluation using community-based benchmarks and the BERT maturity classifier identified 510 studies that met “device-into-practice” criteria, all of which featured validation in real-world clinical settings [21–23]. All included studies were published in English and showed a marked increase in publication volume over time, from one study in 2000–140 in 2023 (S2 Table). After excluding 339 studies with incomplete sociodemographic or performance data, the final analytic cohort comprised 171 studies including 209,772 patients evaluated by mature medical AI models (Fig 1).

### Model development and clinical applications

**Fig 2:**
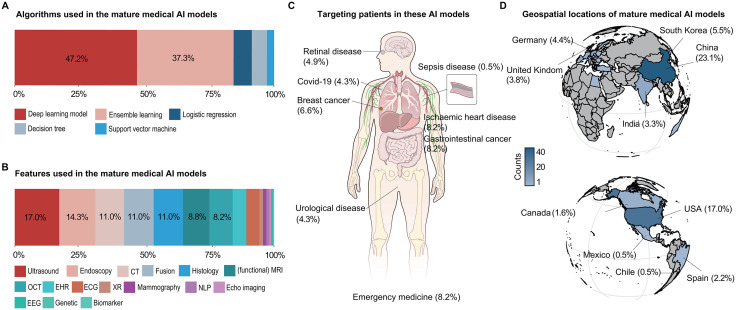
Characteristics of included mature medical AI models. **(A)** Distribution of algorithms. **(B)** Distribution of input features utilized by the included models. Abbreviations for input features are detailed in S3 Table. **(C)** Distribution of diseases among patients using mature AI models. Illustration from NIAID NIH BioArt Source (Licensing: Public Domain; bioart.niaid.nih.gov/bioart/519; bioart.niaid.nih.gov/bioart/55). **(D)** Geospatial distribution of model development, based on the final corresponding author’s institutional affiliation. Basemap data: Natural Earth (public domain, https://www.naturalearthdata.com), via R package “maps”.

Deep learning algorithms were the predominant development approach (47.2% of models), followed by ensemble learning methods (37.3%) (Fig 2A). Most models (81.1%) relied on inputs from diagnostic modalities such as ultrasound, endoscopy, computed tomography, magnetic resonance imaging, optical coherence tomography, and histology, representing a total of 182 modality-specific instances across the included studies (Fig 2B and S3 Table). Clinical applications included emergency medicine, sepsis diagnosis, oncology (e.g., breast and gastrointestinal cancers), and cardiovascular diseases (Fig 2C and S4 Table). Model development was geographically concentrated: China, the U.S., South Korea, and Germany together accounted for more than 50% of studies (Fig 2D and S5 Table).

### Patient demographic characteristics

**Fig 3:**
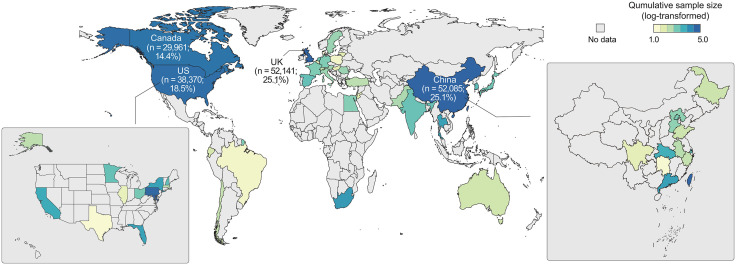
Geographic distribution of participants in studies on mature medical AI models. Basemap data: Natural Earth (public domain, https://www.naturalearthdata.com), via R package “maps”.

Pronounced demographic disparities were observed across the 209,772 patients from 171 studies (Fig 3). Among the 164 studies reporting geographic information (including 207,741 patients), the vast majority of patient cohorts (studies) were drawn from 33 countries, with concentrations in China (n = 47; 28.7%), the U.S. (n = 31; 18.9%), the United Kingdom (n = 11; 6.7%), and South Korea (n = 10; 6.1%) (S6 Table). By contrast, only 2,031 patients (seven studies) were drawn from multi-country cohorts. Dagum–Gini analysis showed extreme global inequality in patient distribution (coefficient = 0.97; *P* < .001 based on 10,000 permutation tests) [20].

When stratified by national income levels, 95.1% of patient cohorts were from high-income (62.2%) or upper-middle-income (32.9%) countries, and 4.9% were from lower-middle-income countries, with no representation from low-income countries [24]. Within leading contributor nations such as China and the U.S., samples were predominantly drawn from affluent regions or cities. Of the 83 studies reporting sex distribution, 63.8% (53 studies) exhibited moderate-to-high sex disparities; among these, 49.1% (26 studies) had male-majority representation. Racial composition was similarly skewed, with White (49.1%) and Asian (42.6%) patients comprising most of the total cohort.

### Model performance and variation across demographic groups

**Fig 4:**
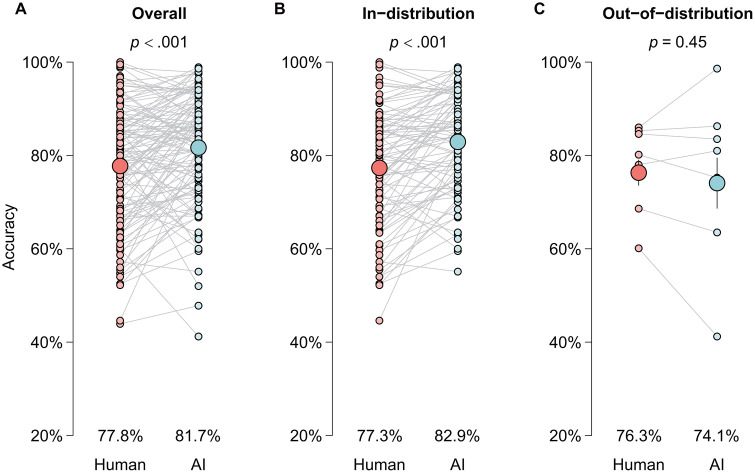
Performance comparisons between mature medical AI models and human practitioners. **(A)** Overall performance comparison between AI models and human practitioners. **(B)** Performance comparison for in-distribution patient groups. **(C)** Performance comparison for out-of-distribution patient groups.

In real-world clinical settings, AI models achieved significantly higher accuracy than human practitioners (81.7% vs. 77.8%; *P* < .001; Fig 4A). This advantage persisted in in-distribution evaluations—within the same country or city in which the model was developed (82.9% vs. 77.3%; *P* < .001; Fig 4B). However, this superiority disappeared in out-of-distribution cohorts (cross-border or cross-city validation), where AI performance became not statistically different from human practitioners (74.1% vs. 76.3%; *P* = .45; Fig 4C). Subgroup analysis showed that deep learning models outperformed shallow learning methods (83.9% vs. 78.9%; *P* = .02) and consistently surpassed human practitioners (83.9% vs. 78.5%, *P* < .001), whereas shallow learning models achieved only parity with human experts (78.9% vs. 76.8%, *P* = .18).

**Fig 5:**
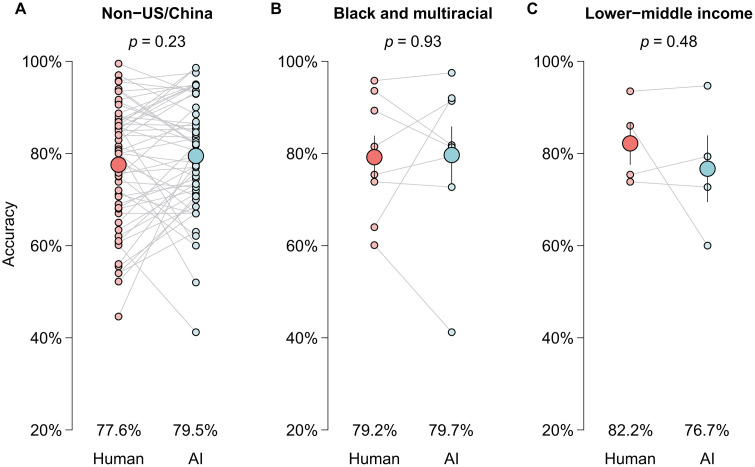
Performance comparisons of AI models to human practitioners in geospatial (A), racial (B), and economic (C) minority groups. Gray points represent mean values displayed at the top of each bar, with solid black lines indicating standard error.

Performance varied systematically across sociodemographic groups. AI models significantly outperformed human practitioners for: (1) patients from geospatial majority regions, defined as China and the U.S.—the two largest single-country contributors in the dataset (83.2% vs. 77.7%; *P* < .001); (2) White and Asian patients (82.0% vs. 78.1%; *P* < .001); and (3) patients from high- or upper-middle-income countries (81.6% vs. 77.5%; *P* < .001). In contrast, for historically underrepresented populations, AI models achieved performance that was not significantly different from human practitioners, including: (1) patients from geospatial minority regions (79.5% vs. 77.6%; *P* = .23; Fig 5A); (2) Black and multiracial patients (79.7% vs. 79.2%; *P* = .93; Fig 5B); and (3) patients from lower-middle-income countries (76.7% vs. 82.2%; *P* = .48; Fig 5C).

Although AI performance was numerically lower in minority than majority groups, these differences were not statistically significant. This “performance parity paradox”—where AI loses its superiority advantage yet maintains potentially non-inferior performance in underrepresented populations—suggests clinical utility in resource-constrained settings despite imperfect generalization.

## Discussion

While sampling biases in AI model development are well recognized, and multiple studies have documented performance degradation when models are applied across geographic or demographic boundaries [6,10], there is limited systematic evidence on which patients actually benefit from mature AI systems in real-world clinical practice. In this context, our findings extend prior work in two key ways. First, we move beyond descriptive accounts by quantitatively demonstrating the extreme concentration of AI benefits among economically advantaged populations, as reflected by a Dagum–Gini coefficient of 0.97. Second, we identify a “performance parity paradox,” in which AI models no longer outperform human practitioners in underrepresented groups but still perform at a level comparable to clinicians.

A primary goal of deploying medical AI is to improve diagnostic accuracy and healthcare quality, particularly in settings where underdiagnosis or misdiagnosis has severe consequences [25–27]. However, our results show that most studies identified from the database do not evaluate prospective, real-world, mature AI models. In this sense, our filtering process functions as a meta-critique of the field: despite rapid growth in clinical AI publications, the literature offers a surprisingly small and fragmented evidence base for practice-ready systems with sufficiently transparent reporting to enable meaningful comparisons. This suggests that the central bottleneck in clinical AI translation lies less in the number of models developed and more in the rigor and completeness with which mature systems are evaluated and reported in real-world settings [28]. Moreover, most AI models approved for real-world use are concentrated in a narrow set of conditions with relatively homogeneous biomarkers—such as emergency medicine, sepsis, and cancer diagnostics on CT, MRI, and genomics—in which human practitioners are already highly proficient [29–31]. Consistent with this observation, several meta-analyses report that market-approved AI models are generally comparable or only slightly superior to junior specialists [32–34]. By contrast, for rare diseases or disorders without established diagnostic standards (e.g., certain psychiatric or autoimmune conditions), AI models have shown more pronounced advantages over clinicians [35–38]. Yet even for these dominant clinical conditions, benefits typically depend on costly radiological infrastructure, thereby perpetuating AI-related health-economic inequalities [39–41]. Together, these observations suggest a misalignment between the areas where AI is currently deployed and the areas where enhanced diagnostic tools are most needed.

Our findings also contrast with certain recent assessments of AI clinical performance. Takita et al. (2025) reported that generative AI models achieved only 52.1% diagnostic accuracy, with no superiority to physicians, whereas we observed an overall accuracy of 81.7% for mature, specialized AI systems [11]. We attribute this discrepancy to differences in the systems evaluated: our dataset primarily captures deployed, task-specific models that have passed regulatory review and been implemented in clinical workflows, whereas Takita et al. examined generative large language models (LLMs). Within our cohort, algorithmic sophistication emerged as a key determinant of performance: only deep learning models significantly outperformed practitioners, while shallow learning approaches did not. Consequently, our performance estimates may not generalize directly to emerging LLM-based clinical applications, underscoring that deployed, task-specific designs and algorithmic sophistication remain central to achieving superior model performance in practice. In parallel, our high performance figures are consistent with Han et al. (2024), who reported that 81% of randomized controlled trials reported positive endpoints; however, our real-world deployment data reveal a critical limitation: these benefits are highly concentrated in specific populations, exposing a structural gap between trial success and equitable clinical translation [13].

The underrepresentation of economically disadvantaged populations in AI development is widely recognized as a major threat to generalizability. While previous analyses of FDA-approved AI devices and insurance data have shown that AI deployments cluster in economically advantaged regions [6,42], these studies rarely examine patient demographics at a granular level [43,44] or address the sociodemographic implications of real-world use [43]. Our study addresses this gap by providing quantitative evidence of a digital divide in patient access to AI benefits in routine clinical settings [45]. Specifically, we document substantial geospatial, sex/gender, racial, and economic disparities among patients who benefit from mature models, with use heavily concentrated in high-income countries and particularly among White and Asian patients in China and the U.S. These findings indicate that monitoring technical performance alone is insufficient; detailed tracking of sociodemographic characteristics is essential to inform policies that prioritize fairness, equity, and diversity in AI deployment, including post-approval evaluations explicitly targeting underrepresented groups [46,47].

Although our results confirm that AI models generally outperform human practitioners, these gains are disproportionately observed among sociodemographic majorities or in contexts that rely on costly algorithms and equipment. While algorithmic biases during development have been extensively discussed, our results provide systematic evidence that these biases persist into the deployment phase, even for models already approved for clinical use. This persistence underscores the need for rigorous post-approval monitoring and real-world performance evaluations to ensure model trustworthiness and efficacy across diverse patient populations [48–50]. Yet, despite calls from researchers and regulators, such post-market evaluations remain rare in practice [51,52]. Without them, there is a substantial risk of extrapolating performance metrics derived from privileged patients to disadvantaged groups—an oversight that may erode trust and further entrench global health disparities [53–56].

The “performance parity paradox” we identify reframes how AI bias in healthcare should be understood, challenging the binary view that any decrease in AI performance constitutes failure. Unlike prior work documenting clear performance degradation in minority populations—for example, Cau et al. (2025), who found evidence of bias in 82% of cardiovascular AI studies [10]—we observe that mature AI models often retain human-equivalent performance even when their superhuman advantage disappears. This suggests that bias in mature systems may manifest as a regression to the human baseline rather than outright incompetence. In underserved regions with specialist shortages and diagnostic delays, particularly in low- and middle-income countries, an AI model that performs “only” at the level of a competent practitioner can still provide substantial value as a scalable diagnostic safety net or triage tool [57,58]. Rather than treating loss of algorithmic superiority in out-of-distribution performance as failure, we propose viewing it as retained clinical utility. Consequently, and consistent with Yang et al.’s (2024) findings on transfer learning, the pragmatic path forward is not to wait for universally perfect models, but to embrace local optimization strategies that validate and adapt these “human-level” tools to meet specific regional needs [7].

From this perspective, even when AI does not yield statistically superior gains for minority or economically disadvantaged groups, its ability to deliver results that are not significantly worse than human practitioners remains clinically meaningful [45,59]. This supports prioritizing optimization for local contexts rather than exclusively pursuing global generalizability [3,60]. While the benefits of mature medical AI models in real-world clinical settings may currently be concentrated within a narrow demographic, these systems can still provide valuable, comparable support to human practitioners when deployed thoughtfully. However, such “comparable performance” must be interpreted cautiously because human benchmarks themselves vary systematically with socioeconomic conditions [61]. In resource-limited settings, systemic constraints—such as reduced specialist density, practitioner fatigue, and suboptimal equipment—often suppress the human baseline [62,63]. Thus, our finding that AI matches humans in these environments may reflect alignment with a constrained standard of care rather than true clinical excellence. Future work must therefore assess whether this level of accuracy satisfies absolute thresholds for safe deployment, independent of potentially lowered local benchmarks.

Our quantitative characterization of access disparities advances the field beyond general calls for inclusivity in prior reviews [14]. The extreme concentration observed in our analysis (95.1% of patient cohorts from high- or upper-middle-income countries) strongly suggests that current regulatory and market mechanisms are failing to democratize AI benefits. Instead of focusing solely on bias mitigation during model development, our findings point to the need for structural interventions in deployment and access. We propose a three-pronged strategy. First, for technical adaptation, region-specific optimization strategies such as transfer learning and federated learning should be used to minimize performance degradation across diverse populations [64]. Second, rigorous local validation should be mandated before deployment so that “non-inferiority” is judged against objective clinical standards rather than suppressed local baselines [65]. Third, regulatory frameworks must evolve beyond simple accuracy metrics to include explicit incentives for inclusivity, such as requiring minimum demographic diversity thresholds in training datasets for market approval [66]. Together, these measures offer a path to transform AI from a commercially concentrated asset into a broadly accessible clinical resource.

### Limitations

Our study has several limitations. First, the mature medical AI models analyzed are primarily identified through scientific publications, potentially overlooking unpublished models currently used in clinical settings. This reliance on the published literature may also introduce regional publication bias (e.g., studies from high-income countries may report both successes and failures, whereas studies from low- and middle-income countries may selectively publish positive results), potentially underestimating AI–human gaps in underserved settings. Furthermore, our geographic and economic comparisons are confounded by healthcare system quality. Regions with limited resources often have fewer specialists, less training infrastructure, and higher practitioner workloads. Thus, findings that AI is comparable to humans in these settings may indicate AI matching a suboptimal baseline rather than achieving adequate clinical performance. Second, our sample is likely enriched for task-specific, regulated systems and may underrepresent emerging LLM-based or generative AI tools; therefore, the findings may not directly generalize to these rapidly evolving paradigms. Third, patients included in the studies were prospectively selected for testing within hospitals and medical centers. This clinical setting, though distinct from experimental conditions typical of clinical trials, may introduce sampling biases tied to the practices of specific medical and research institutions. Fourth, the included studies span nearly 24 years (2000–2024), during which AI capabilities evolved substantially, so temporal pooling may blur differences between early-generation systems and current state-of-the-art models. Finally, the absence of data from manufacturers and electronic medical record providers limits our ability to assess model performance across diverse clinical environments; additionally, our analyses cannot fully account for non-independence when the same models or widely used datasets are evaluated across multiple studies, potentially inflating the effective sample size. Nonetheless, validation study populations likely serve as a reasonable proxy for clinical access patterns, given the strong correlation between research infrastructure, economic resources, and clinical AI deployment capabilities. Regions and institutions with the capacity to conduct rigorous AI validation studies typically also lead in clinical AI implementation.

## Conclusions

Despite the rapid growth of medical AI models in areas such as sepsis diagnosis and cancer detection, their deployment in real-world settings remains heavily skewed, favoring patients from high-income countries and certain sociodemographic groups, particularly Asian and White populations. We identify a critical pattern in which these models, although outperforming human practitioners in many cases, lose their performance advantage for socioeconomically disadvantaged groups, including those in lower-middle-income countries or minority communities. Consequently, enhancing inclusivity and mandating rigorous post-market evaluations are essential to bridge this gap.

Crucially, while AI models maintain performance not significantly worse than human practitioners, we emphasize that whether this represents adequate performance requires validation against objective standards of care rather than constrained local baselines. Ultimately, this capacity of mature medical AI systems to sustain human-level accuracy in underserved regions underscores their potential to provide scalable diagnostic support and drive more equitable healthcare innovation, provided that deployment is guided by rigorous, context-aware validation.

## Code availability

Custom analysis scripts used to calculate Dagum–Gini coefficients, run statistical analyses, and generate figures are available at https://www.scidb.cn/s/INfQ7r.

## Supporting information

S1 Method Detailed methodology of the BERT-PubMed classifiers used for study screening and characterization. (DOCX)

S1 Fig Flowchart of the search and inclusion process for studies on mature medical AI models following PRISMA guidelines. (DOCX)

S1 Table Prompt for the GPT-aided categorization of AI models. (DOCX)

S2 Table Distribution of publication years of included studies. (DOCX)

S3 Table Distribution of input features used in the mature medical AI models. (DOCX)

S4 Table Distribution of disease subspecialty in the mature medical AI models. (DOCX)

S5 Table Distribution of affiliated institutions of the final corresponding author. (DOCX)

S6 Table Geographical distribution of patient cohorts (studies) in the mature medical AI models. (DOCX)
