# Supplementary material for: Characterizing patients who benefit from mature medical AI models in real-world clinical applications
Source: PLOS Digit Health. 2026 Mar 20;5(3):e0001283. doi: 10.1371/journal.pdig.0001283 (PMC13004356; doi:10.1371/journal.pdig.0001283)
Supplement: S1 Method — (DOCX) [file pdig.0001283.s001.docx]

**S1 Method**

**BERT-PubMed inclusion classifier**

To automatically screen eligible studies, an inclusion classifier was fine-tuned from the pretrained BERT-PubMed model (Madabushi et al., 2020), which has demonstrated strong performance on biomedical language tasks. Using the AdamW optimizer, a binary classifier was trained to determine whether a paper reports AI models that provide predictive, diagnostic, or otherwise quantitatively informative outputs for patient care. An additional question was added to enhance classification accuracy: “*Does this model’s output have a direct, actionable effect on patient care, by providing information to a healthcare provider, patient, or automated system?*” Studies labeled “Yes” by the classifier were initially included for further review. The resulting fine-tuned model is referred to as the BERT-PubMed Inclusion Classifier.

The training dataset for this classifier, curated by Zhang et al. (2022), consisted of 4,000 PubMed abstracts (1998–2020) with manually assigned labels (e.g., meeting vs. not meeting inclusion criteria), using a 9:1 train–test split. To assess generalizability, the authors tested the classifier on an additional 1,000 manually labeled abstracts published after September 2021. Sensitivity was further evaluated using a separate dataset of 446 manually labeled abstracts from a systematic review (Aggarwal et al., 2021). This final inclusion classifier achieved high performance, with both accuracy and sensitivity exceeding 0.98. Training and testing datasets are available at <https://github.com/whizzlab/health_ai_training>.

**BERT-PubMed maturity classifier**

Beyond relevance to patient care, AI models included in this study were required to be at the “device-into-practice” maturity stage, indicating regulatory approval or deployment in real-world clinical settings. Community-defined benchmarks (Lavin et al., 2021; Sadiq et al., 2021; Zhang et al., 2022) conceptualize AI maturity as spanning from the “math-into-algorithm” stage (developmental, without real-world data) to the “device-into-practice” stage (real-world clinical deployment).

Zhang et al. (2022) fine-tuned a second classifier, the BERT-PubMed Maturity Classifier, to categorize studies identified by the inclusion classifier as mature or immature. The training dataset contained 2,500 PubMed abstracts (1998–2020) labeled by maturity level. An additional set of 2,494 abstracts published after September 2021 was used to test generalizability. Sensitivity was evaluated using an independent set of 83 manually labeled abstracts from another systematic review (Nagendran et al., 2020). The maturity classifier showed high performance, with accuracy >0.99 and sensitivity >0.97.

**BERT-PubMed multi-label classifier**

Once eligibility was confirmed by the inclusion and maturity classifiers, Zhang et al. (2022) used a named-entity recognition (NER) NLP model (SparkNLP based on work by Chiu and Nichols, 2016) combined with a dictionary-based text recognition layer to automatically extract model characteristics, including clinical specialty, subspecialty or disease, type of algorithm used, and type of data input into models. For online deployment, manually validated NER labels were used to train a BERT-PubMed multi-label classifier so that abstracts could receive more than one label in each class. Training for the inclusion classifier was initiated on 4,000 manually labeled abstracts.

**eReferences**

1. Aggarwal, R., Sounderajah, V., Martin, G., Ting, D. S. W., Karthikesalingam, A., King, D., Ashrafian, H., & Darzi, A. (2021). Diagnostic accuracy of deep learning in medical imaging: a systematic review and meta-analysis. npj Digital Medicine, 4(1), 65.
2. Chiu, J. P., & Nichols, E. (2016). Named entity recognition with bidirectional LSTM-CNNs. Transactions of the Association for Computational Linguistics, 4, 357-370.
3. Lavin, A., Gilligan-Lee, C. M., Visnjic, A., Ganju, S., Newman, D., Ganguly, S., Lange, D., Baydin, A. G., Sharma, A., Gibson, A., Zheng, S., Xing, E. P., Mattmann, C., Parr, J., & Gal, Y. (2022). Technology readiness levels for machine learning systems. Nature Communications, 13(1), 6039.
4. Madabushi, H. T., Kochkina, E., & Castelle, M. (2020). Cost-sensitive BERT for generalisable sentence classification with imbalanced data. arXiv preprint arXiv:2003.11563.
5. Sadiq, R. B., Safie, N., Abd Rahman, A. H., & Goudarzi, S. (2021). Artificial intelligence maturity model: a systematic literature review. PeerJ Computer Science, 7, e661.
6. Zhang, J., Whebell, S., Gallifant, J., Budhdeo, S., Mattie, H., Lertvittayakumjorn, P., Del Pilar Arias Lopez, M., Tiangco, B. J., Gichoya, J. W., Ashrafian, H., Celi, L. A., & Teo, J. T. (2022). An interactive dashboard to track themes, development maturity, and global equity in clinical artificial intelligence research. The Lancet Digital Health, 4(4), e212–e213.
