# Supplementary material for: Characterizing patients who benefit from mature medical AI models in real-world clinical applications
Source: PLOS Digit Health. 2026 Mar 20;5(3):e0001283. doi: 10.1371/journal.pdig.0001283 (PMC13004356; doi:10.1371/journal.pdig.0001283)

**S1 Fig.** Flowchart of the search and inclusion process for studies on mature medical AI models following PRISMA guidelines


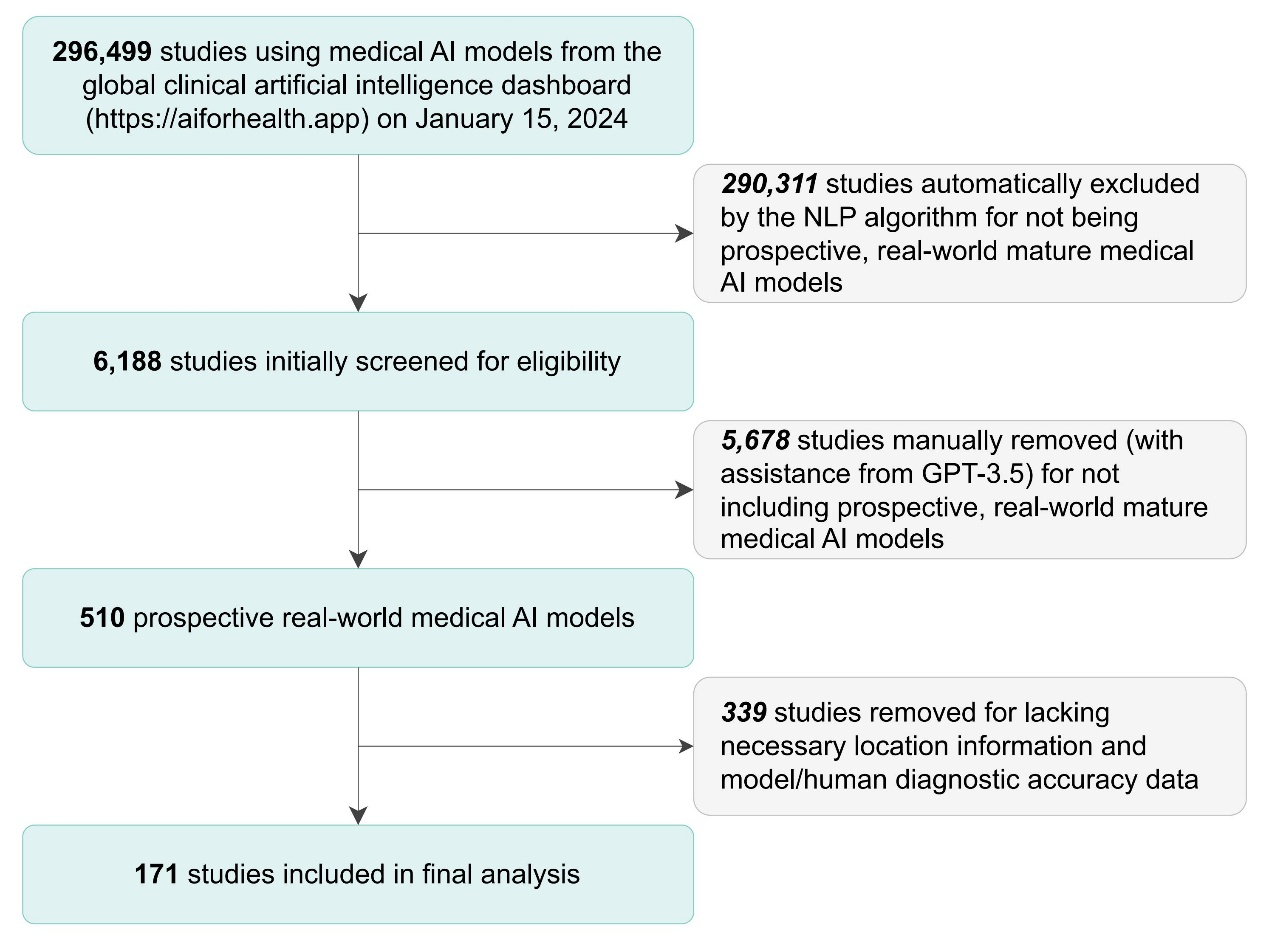

Supplement: S1 Fig — (DOCX) [file pdig.0001283.s002.docx]
