# Supplementary material for: Characterizing patients who benefit from mature medical AI models in real-world clinical applications
Source: PLOS Digit Health. 2026 Mar 20;5(3):e0001283. doi: 10.1371/journal.pdig.0001283 (PMC13004356; doi:10.1371/journal.pdig.0001283)
Supplement: S1 Table — (DOCX) [file pdig.0001283.s003.docx]

**S1_Table. Prompt for the GPT-aided categorization of AI models**

|  | **Prompt** |
| --- | --- |
| **#1** | Hi, you are a professional expert in medical AI. Could you help me identify whether this paper can be categorized as "comparative AI" that describes model testing against a non-AI, existing, gold standard (analogous to a comparative clinical study), "prospective real-world validation AI" that describes deployment for validation in a prospective real-world environment, or "none of these", based on the paper abstract that I will provide to you? |
| **#2** | Here is the abstract “ ... ”. Please categorize it as "comparative AI," "prospective real-world validation AI", or "none of these", and provide reasons. |
