# Supplementary material for: Characterizing patients who benefit from mature medical AI models in real-world clinical applications
Source: PLOS Digit Health. 2026 Mar 20;5(3):e0001283. doi: 10.1371/journal.pdig.0001283 (PMC13004356; doi:10.1371/journal.pdig.0001283)
Supplement: S2 Table — (DOCX) [file pdig.0001283.s004.docx]

**S2_Table. Distribution of publication years of included studies.***Note:* Publication year could not be determined for 58 of the 510 included studies.

| **Publication year** | **No.** | **Proportion, %** |
| --- | --- | --- |
| 2000 | 1 | 0.22 |
| 2005 | 1 | 0.22 |
| 2006 | 1 | 0.22 |
| 2007 | 1 | 0.22 |
| 2012 | 1 | 0.22 |
| 2013 | 3 | 0.66 |
| 2014 | 1 | 0.22 |
| 2015 | 3 | 0.66 |
| 2016 | 3 | 0.66 |
| 2017 | 2 | 0.44 |
| 2018 | 15 | 3.32 |
| 2019 | 30 | 6.64 |
| 2020 | 50 | 11.06 |
| 2021 | 79 | 17.48 |
| 2022 | 117 | 25.88 |
| 2023 | 140 | 30.97 |
| 2024 | 4 | 0.88 |
