# Supplementary material for: Characterizing patients who benefit from mature medical AI models in real-world clinical applications
Source: PLOS Digit Health. 2026 Mar 20;5(3):e0001283. doi: 10.1371/journal.pdig.0001283 (PMC13004356; doi:10.1371/journal.pdig.0001283)
Supplement: S3 Table — (DOCX) [file pdig.0001283.s005.docx]

**S3_Table. Distribution of input features used in the mature medical AI models**

| **Input feature** | **No.** | **Proportion, %** |
| --- | --- | --- |
| Ultrasound (US) | 31 | 17.0 |
| Endoscopy images (ENDO) | 26 | 14.3 |
| Computed Tomography (CT) | 20 | 11.0 |
| Fusion feature | 20 | 11.0 |
| Histology (HISTO) | 20 | 11.0 |
| Magnetic Resonance Imaging (MRI) | 16 | 8.8 |
| Optical Coherence Tomography (OCT) | 15 | 8.2 |
| Electronic Health Record (EHR) | 9 | 4.9 |
| Electrocardiography (ECG) | 8 | 4.4 |
| X-rays (XR) | 5 | 2.7 |
| Mammography (MAMM) | 4 | 2.2 |
| Natural Language Processing (NLP) | 3 | 1.6 |
| Echocardiography (ECHO) | 2 | 1.1 |
| Electroencephalography (EEG) | 1 | 0.5 |
| Genome | 1 | 0.5 |
| Biomarker | 1 | 0.5 |
