# Supplementary material for: Characterizing patients who benefit from mature medical AI models in real-world clinical applications
Source: PLOS Digit Health. 2026 Mar 20;5(3):e0001283. doi: 10.1371/journal.pdig.0001283 (PMC13004356; doi:10.1371/journal.pdig.0001283)
Supplement: S4 Table — (DOCX) [file pdig.0001283.s006.docx]

**S4_Table. Distribution of disease subspecialty in the mature medical AI models**

| **Disease subspecialty** | **No.** | **Proportion, %** |
| --- | --- | --- |
| Emergency medicine | 15 | 8.2 |
| Gastrointestinal cancer | 15 | 8.2 |
| Ischemic heart disease | 15 | 8.2 |
| Breast cancer | 12 | 6.6 |
| Retinal | 9 | 4.9 |
| COVID-19 | 8 | 4.4 |
| Urology | 8 | 4.4 |
| Intensive Care | 7 | 3.8 |
| Prostate cancer | 5 | 2.7 |
| Pneumonia | 4 | 2.2 |
| Skin cancer | 3 | 1.6 |
| Lung cancer | 3 | 1.6 |
| Stroke | 3 | 1.6 |
| Heart failure | 3 | 1.6 |
| Brain cancer | 2 | 1.1 |
| Hepatic cancer | 2 | 1.1 |
| Gynae-oncology | 2 | 1.1 |
| Sepsis | 1 | 0.5 |
| Epilepsy | 1 | 0.5 |
| Arrhythmia | 1 | 0.5 |
