# Supplementary material for: Characterizing patients who benefit from mature medical AI models in real-world clinical applications
Source: PLOS Digit Health. 2026 Mar 20;5(3):e0001283. doi: 10.1371/journal.pdig.0001283 (PMC13004356; doi:10.1371/journal.pdig.0001283)
Supplement: S5 Table — (DOCX) [file pdig.0001283.s007.docx]

**S5_Table. Distribution of affiliated institutions of the final corresponding author**

| **Country, region, or area** | **No.** | **Proportion, %** |
| --- | --- | --- |
| China | 42 | 23.1 |
| United States | 31 | 17.0 |
| South Korea | 10 | 5.5 |
| Germany | 8 | 4.4 |
| Taiwan | 7 | 3.8 |
| United Kingdom | 7 | 3.8 |
| India | 6 | 3.3 |
| Japan | 5 | 2.7 |
| France | 4 | 2.2 |
| Netherlands | 4 | 2.2 |
| Spain | 4 | 2.2 |
| Canada | 3 | 1.6 |
| Jordan | 3 | 1.6 |
| Israel | 2 | 1.1 |
| Singapore | 2 | 1.1 |
| Switzerland | 2 | 1.1 |
| Australia | 1 | 0.5 |
| Austria | 1 | 0.5 |
| Belgium | 1 | 0.5 |
| Brazil | 1 | 0.5 |
| Chile | 1 | 0.5 |
| Egypt | 1 | 0.5 |
| Hungary | 1 | 0.5 |
| Ireland | 1 | 0.5 |
| Italy | 1 | 0.5 |
| Lithuania | 1 | 0.5 |
| Mexico | 1 | 0.5 |
| Poland | 1 | 0.5 |
| Romania | 1 | 0.5 |
| Sweden | 1 | 0.5 |
| Turkey | 1 | 0.5 |
