# Supplementary material for: Characterizing patients who benefit from mature medical AI models in real-world clinical applications
Source: PLOS Digit Health. 2026 Mar 20;5(3):e0001283. doi: 10.1371/journal.pdig.0001283 (PMC13004356; doi:10.1371/journal.pdig.0001283)
Supplement: S6 Table — (DOCX) [file pdig.0001283.s008.docx]

**S6_Table. Geographical distribution of patient cohorts (studies) in the mature medical AI models**

| **Country, region, or area** | **No.** | **Proportion, %** |
| --- | --- | --- |
| China | 47 | 28.7 |
| United States | 31 | 18.9 |
| United Kingdom | 11 | 6.7 |
| South Korea | 10 | 6.1 |
| Germany | 6 | 3.7 |
| Japan | 6 | 3.7 |
| Netherlands | 5 | 3.0 |
| France | 4 | 2.4 |
| India | 4 | 2.4 |
| Spain | 4 | 2.4 |
| Canada | 3 | 1.8 |
| Israel | 3 | 1.8 |
| Sweden | 3 | 1.8 |
| Switzerland | 3 | 1.8 |
| Austria | 2 | 1.2 |
| Belgium | 2 | 1.2 |
| Italy | 2 | 1.2 |
| Pakistan | 2 | 1.2 |
| Singapore | 2 | 1.2 |
| Australia | 1 | 0.6 |
| Brazil | 1 | 0.6 |
| Chile | 1 | 0.6 |
| Denmark | 1 | 0.6 |
| Ecuador | 1 | 0.6 |
| Egypt | 1 | 0.6 |
| Hungary | 1 | 0.6 |
| Jordan | 1 | 0.6 |
| Lithuania | 1 | 0.6 |
| Poland | 1 | 0.6 |
| Romania | 1 | 0.6 |
| South Africa | 1 | 0.6 |
| Thailand | 1 | 0.6 |
| Turkey | 1 | 0.6 |
